# Carbon farming strategies for mediterranean agriculture: the role of biochar in climate-smart agroecosystems

**DOI:** 10.1007/s44297-026-00081-8

**Published:** 2026-07-14

**Authors:** Daniele Borgatti, Emanuele Radicetti, Roberto Mancinelli, Lorenzo Coluccia, Mohamed Allam, Aftab Jamal, Zainul Abideen, Muhammad Ahsan, Mortadha Ben Hassine

**Affiliations:** 1 Department of Chemical, Pharmaceutical and Agricultural Sciences (DOCPAS), University of Ferrara, 44121 Ferrara, Italy; 2 Department of Agriculture and Forest Sciences (DAFNE), University of Tuscia, 01100 Viterbo, Italy; 3 Department of Agronomy, Faculty of Agriculture, Assuit University, Assuit, 71526 Egypt; 4 Department of Soil and Environmental Sciences, Faculty of Crop Production Sciences, The University of Agriculture, Peshawar, 25130 Pakistan; 5 Dr. Muhammed Ajmal Khan Institute of Sustainable Halophyte Utilization, University of Karachi, Karachi, 75270 Pakistan; 6 Sharjah Seed Bank and Herbarium, University of Al Dhaid, Sharjah, United Arab Emirates; 7 Department of Horticultural Sciences, The Islamia University of Bahawalpur, Bahawalpur, 63100 Pakistan

**Keywords:** Biochar, Carbon sequestration, Mediterranean region, Greenhouse gas emissions, Climate change mitigation, Sustainable agriculture, Crop productivity

## Abstract

Mediterranean agriculture is increasingly constrained by climate change–driven stresses, including rising temperatures, intensified drought, and soil organic matter depletion, all of which threaten crop health and yield stability. Carbon farming has emerged as a strategy to integrate climate mitigation with agricultural resilience, and biochar represents a distinctive tool within this framework due to its capacity for long-term carbon sequestration and soil modification. This review synthesizes peer-reviewed studies published between 1999 and 2025 to assess the role of biochar in Mediterranean agroecosystems, with a specific focus on crop health outcomes. Across Mediterranean systems, biochar consistently increases soil organic carbon stocks through the addition of recalcitrant carbon forms and generally reduces nitrous oxide emissions while carbon dioxide emissions remain neutral. However, methane emissions may increase under warm and moist conditions, highlighting the importance of comprehensive greenhouse gas accounting. Biochar improves crop performance primarily when it alleviates limiting soil constraints, particularly in degraded or coarse-textured soils, under water-limited or saline conditions, and in perennial cropping systems. Long-term benefits are most evident in tree crops and vineyards, and legumes often show positive responses linked to enhanced nutrient availability and rhizosphere functioning. In contrast, cereal and leafy vegetable crops exhibit more variable responses, including neutral or negative effects under non-limiting conditions. Overall, biochar is most effective when applied selectively at moderate rates (approximately 10–30 Mg ha⁻^1^) and integrated with complementary climate-smart practices. Future research should prioritize long-term, crop-centered assessments and methane mitigation strategies to support context-specific biochar deployment in Mediterranean agriculture.

## Introduction

Nowadays, agroecosystems in the Mediterranean area are considered a climate change area subjected to desertification processes [1] and are facing multiple environmental challenges, including global warming [2], biodiversity loss [3], soil erosion [4, 5], and population growth [6]. Projected temperature increases of approximately 0.45 °C per decade, coupled with declining precipitation and intensifying drought events, are accelerating soil degradation processes that threaten the viability of traditional farming systems across the region [7]. Agricultural soils under Mediterranean agroecosystems are generally characterized by low organic matter content, high carbonate levels, and susceptibility to erosion; furthermore, they are experiencing further deterioration through intensive cultivation, leading to reduced fertility, diminished water-holding capacity, and increased vulnerability to desertification [4, 5, 8]. Simultaneously, Mediterranean agriculture must meet growing food demands from expanding populations while reducing its environmental footprint, particularly greenhouse gas (GHG) emissions that contribute to the climate crisis [6]. The need to maintain productivity while enhancing environmental sustainability requires innovative and sustainable approaches that can simultaneously address climate change mitigation, ecosystem restoration, and agricultural resilience.

Recently, carbon farming has emerged as a strategic approach to harmonize the need for food demands by developing agricultural practices that actively remove atmospheric carbon dioxide and store it in agroecosystems while improving ecosystem functions [9]. The concept encompasses diverse management interventions, including (1) conservation tillage that minimizes soil disturbance, cover cropping that maintains living roots; (2) agroforestry systems that integrate trees with crops, optimized nutrient management that reduces fertilizer-related emissions, and (3) the application of stabilized organic amendments such as compost and biochar [8]. These agronomical practices operate by means of complementary mechanisms that act for enhancing biological carbon inputs through increased photosynthesis and biomass production [10], reducing carbon losses by protecting existing soil organic matter (SOM) from decomposition [11], and introducing recalcitrant carbon forms that resist degradation over time [12]. Under this context, biochar application represents a promising strategy for Mediterranean agroecosystems due to its characteristics that address multiple challenges simultaneously. Biochar is produced through the pyrolysis process that causes a thermal decomposition of organic materials under oxygen-limited conditions. By means of pyrolysis, several feedstocks, including agricultural residues, forestry waste, and animal manure, are converted into a stable, carbon-rich material [11, 12]. Pyrolysis occurs in a low-oxygen environment at a high temperature and can be classified into slow pyrolysis, fast pyrolysis, and gasification, depending on processing conditions such as temperature that can range from 300 °C to 1000 °C [12]. The pyrolysis process operates across different temperature ranges and residence times, creating biochar characterized by different physicochemical properties tailored to specific applications [13]. This production pathway aligns with circular economy principles by transforming waste streams into valuable agricultural inputs while simultaneously sequestering atmospheric carbon in a stable structure [14, 15]. Due to its characteristics, biochar differs from conventional organic amendments as stable material; indeed, organic amendments, after their application, are subjected to microorganisms’ actions that decompose the organic substances gradually into minerals; conversely, the carbon content in biochar persists in soils, offering an efficient atmospheric carbon removal rather than temporary storage [16].

The mechanistic basis for biochar’s multifunctional role in climate-smart Mediterranean agriculture affects the chemical, physical, and microbiological processes [17], enhancing soil fertility [18] and crop productivity [19]. Indeed, biochar porous architecture can enhance soil structure, reduce bulk density, and improve water-holding capacity, particularly relevant in Mediterranean agroenvironmental conditions where water availability is limited [20]. In addition, it is well known that biochar can increase soil pH, cation exchange capacity (CEC), and reduce nutrient leaching by adsorbing key ions such as ammonium, nitrate, and phosphate [21]. Additionally, biochar mitigates ammonia volatilization by absorbing ammonium/ammonia [21] and enhances nitrification processes [22]. Biochar also provides habitat for beneficial microorganisms, and it has been observed to have a capacity to suppress certain soil-borne pathogens and may enhance mycorrhizal associations that improve plant nutrient acquisition [23, 24]. By modifying soil nutrient cycles, biochar ultimately improves crop production [25]. Moreover, biochar enhances crop resilience to biotic and abiotic stresses, thereby improving overall productivity [26]. Beyond these soil improvements, biochar application contributes directly to climate change mitigation by transferring atmospheric carbon into long-term terrestrial storage while potentially reducing emissions of nitrous oxide and methane from agricultural soils, although effects on these trace gases has shown to be considerably context dependent [27, 28]. Based on these characteristics, biochar represents an innovative solution for enhancing the sustainability of agroecosystems due to its potential to convert agricultural waste in an efficient soil amendment [29].

Despite these benefits, the practical implementation of biochar application in agroecosystems in the Mediterranean area remains constrained, inadequately characterized, and poorly understood [30]. The Mediterranean region presents unique environmental conditions—specifically, hot, dry summers coupled with mild, wet winters—that distinguish it from temperate, tropical, or continental zones, where a lot of biochar research has been conducted [31]. The existing literature on biochar in Mediterranean agriculture presents a fragmented and sometimes contradictory picture. While numerous Mediterranean studies document beneficial effects on crop yields, soil carbon accumulation, and water-use efficiency across diverse systems, including cereals, vegetables, and tree crops, others report negligible impacts or even adverse outcomes, such as reduced plant growth in certain species, increased methane emissions under specific soil moisture regimes, nutrient immobilization in particular soil types, and negative effects on beneficial soil fauna [32–34]. However, previous reviews have not systematically examined how these factors operate specifically within Mediterranean contexts, nor have they provided clear guidance on when and how biochar should be applied in the Mediterranean area. Carbon credit markets, potentially crucial financing mechanisms for biochar implementation, remain uncertain across Mediterranean countries, with pricing trajectories difficult to monitor and inconsistent policy support frameworks [35]. This implementation gap between research evidence and agricultural practice represents a critical barrier to realizing biochar’s potential contribution to climate-smart Mediterranean agriculture.

Recent brief bibliographic research on the main topics related to carbon farming in the Mediterranean basin demonstrated an increment of scientific researchers’ interest in applying biochar as a climate-smart strategy (Table 1) [36–45]. However, this knowledge should be elucidated to farmers, who are the real actors in the Mediterranean farms.

**Table 1:** Top 10 highly cited papers presenting results of experiments based on biochar in the Mediterranean region published from 1999 to 2026, present in the database of Scopus.*

No	Ref	Title	Source name	No. of references cited
1	Graber et al. [36]	Biochar impact on development and productivity of pepper and tomato grown in fertigated soilless media	Plant and Soil	716
2	Angın [37]	Effect of pyrolysis temperature and heating rate on biochar obtained from pyrolysis of safflower seed press cake	Bioresource Technology	660
3	Agrafioti et al. [38]	Biochar production by sewage sludge pyrolysis	Journal of Analytical and Applied Pyrolysis	587
4	Pallarés et al. [39]	Production and characterization of activated carbon from barley straw by physical activation with carbon dioxide and steam	Biomass and Bioenergy	523
5	Fellet et al. [40]	Application of biochar on mine tailings: effects and perspectives for land reclamation	Chemosphere	462
6	Méndez et al. [41]	Effects of sewage sludge biochar on plant metal availability after application to a Mediterranean soil	Chemosphere	450
7	Özçimen and Ersoy-Meriçboyu [42]	Characterization of biochar and bio-oil samples obtained from carbonization of various biomass materials	Renewable Energy	426
8	Agrafioti et al. [43]	Arsenic and chromium removal from water using biochars derived from rice husk, organic solid wastes and sewage sludge	Journal of Environmental Management	416
9	Vaccari et al. [44]	Biochar as a strategy to sequester carbon and increase yield in durum wheat	European Journal of Agronomy	388
10	Kolton et al. [45]	Impact of biochar application to soil on the root-associated bacterial community structure of fully developed greenhouse pepper plants	Applied and Environmental Microbiology	363

^*^Data access till 26 January 2026

This review addresses these critical gaps by providing the first comprehensive synthesis of biochar research specifically within the Mediterranean carbon farming context. The main aims are to (1) synthesize evidence on biochar’s contribution to climate change mitigation in Mediterranean conditions, including carbon sequestration rates, long-term stability, and GHG emission dynamics (carbon dioxide, nitrous oxide, methane); (2) evaluate biochar effects on physical, chemical, and biological soil properties across the Mediterranean area; and (3) assess crop productivity and physiological responses to biochar application in Mediterranean agricultural systems.

## Literature analysis: methodology

The Scopus database was used to collect peer-reviewed articles on biochar research conducted in the Mediterranean region. The literature analysis covered the period from 1999 to 2025 and considered publication year, citation count, country, and application area. The search query was performed in Scopus using TITLE-ABS-KEY (“biochar”) and was limited to peer-reviewed articles published in English. This initial search yielded more than 50,000 documents. The results were subsequently refined by restricting the subject area using the keywords “carbon farming” and “Mediterranean basin.” After further filtering to include only peer-reviewed journal articles, a total of 112 publications were retained for analysis. The dataset was exported to a CSV file format and used as input for bibliometric analysis. VOSviewer software [46] was employed to construct and visualize bibliographic networks. Given the diversity of study designs and objectives, this work adopts a structured narrative review approach rather than a formal systematic review. The aim is to provide a comprehensive and integrative synthesis of current knowledge while identifying key trends and research gaps. Table 1 [36–45] presents a selection of highly cited studies as illustrative examples of influential contributions in the field rather than a systematic subset of the reviewed literature.

## Biochar in mediterranean carbon farming: implications for crop health and climate mitigation

Carbon farming strategies aim to reconcile climate change mitigation with sustained crop productivity by enhancing carbon sequestration while reducing GHG emissions from agricultural systems. In Mediterranean agroecosystems, where soils are often characterized by low organic matter, high carbonate content, and strong seasonal moisture variability, biochar has emerged as a potentially valuable carbon farming tool [8, 19, 47]. Unlike conventional organic amendments, biochar introduces a highly stable form of carbon that persists in soils over long timescales while simultaneously influencing soil processes relevant to crop health and productivity [11, 47].

### Soil carbon sequestration under Mediterranean conditions

**Fig. 1:**
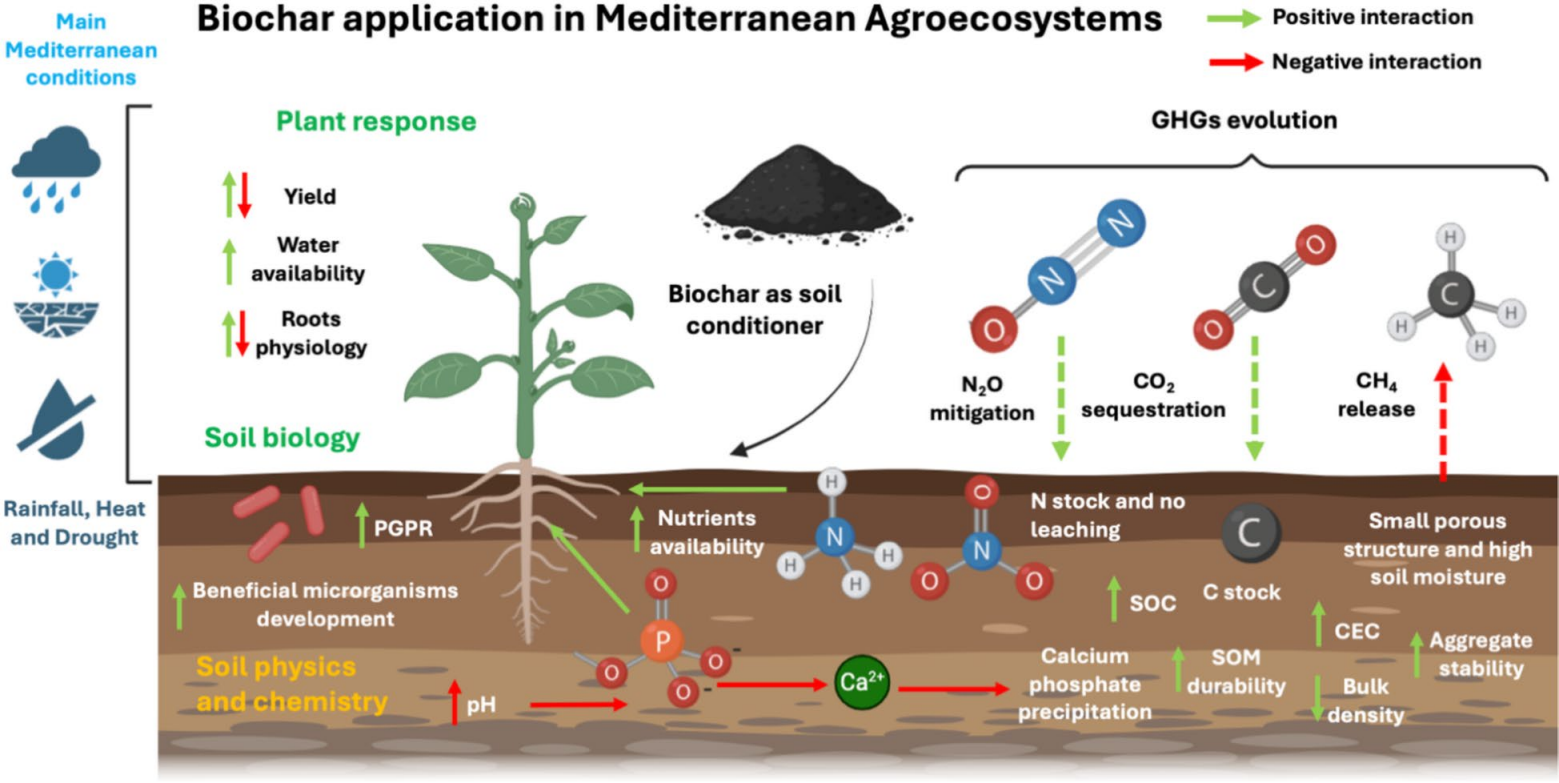
Conceptual framework regarding the role of biochar in climate-smart carbon farming within Mediterranean agroecosystems. Green and red arrows indicate positive and negative interactions, respectively. Abbreviations: C, carbon; Ca^2+^, calcium ion; CEC, cation exchange capacity; CH_4_, methane; CO_2_, carbon dioxide; GHGs, greenhouse gases; H, hydrogen; N, nitrogen; N_2_O, nitrous oxide; O, oxygen; P, phosphorus; PGPR, plant growth-promoting rhizobacteria; SOC, soil organic carbon; SOM, soil organic matter

Biochar contributes directly to soil carbon sequestration through the addition of recalcitrant pyrogenic carbon that resists microbial decomposition, with estimated mean residence times ranging from several centuries to over a millennium, depending on feedstock and pyrolysis conditions [13, 48]. Recent meta-analysis evidence indicates that biochar application increases soil organic carbon (SOC) stocks by an average of approximately 39% at the global scale [8]. Under Mediterranean conditions, available field studies confirm consistent SOC accumulation following biochar application, although reported increases vary depending on soil texture, climatic conditions, and biochar characteristics, reflecting the strong context dependency of carbon sequestration processes in the region. Mediterranean field studies confirm that biochar-derived carbon remains stable under conditions of elevated temperature and pronounced wet–dry cycles. Giannetta et al. [27] demonstrated that biochar-induced increases in SOC pools were not significantly altered by simulated climate change scenarios, suggesting resilience of biochar carbon stocks under future Mediterranean climates (Fig. 1). In addition to direct carbon inputs, biochar can stabilize native SOM through negative priming effects, reducing the mineralization rate of existing SOC [49].

As reported in Fig. 1, biochar plays an important role in enhancing soil carbon stabilization and long-term carbon storage through multiple interacting pathways. This mechanism appears particularly relevant in Mediterranean soils, where rapid organic matter turnover is promoted by high temperatures and long dry periods [50]. In addition, biochar properties vary substantially depending on feedstock type and pyrolysis conditions, particularly temperature. Higher pyrolysis temperatures (e.g., ≥ 600–700 °C) generally produce biochar characterized by increased aromaticity, greater structural stability, higher pH, and larger specific surface area, whereas lower temperatures (e.g., 300–400 °C) result in biochar with a higher content of labile organic compounds and oxygen-containing functional groups, which can enhance short-term nutrient availability and CEC. Feedstock type further influences biochar properties: manure-derived biochar typically exhibits higher nutrient content and ash fractions, whereas lignocellulosic feedstocks produce biochar with higher carbon stability and porosity [13, 51]. Despite these well-established differences, many studies conducted under Mediterranean conditions do not systematically distinguish among biochar types when evaluating soil and crop responses. This lack of standardization contributes to the variability observed in experimental results and limits the comparability of findings across studies. However, highly stable biochar may exhibit reduced short-term effects on soil fertility and crop performance, indicating a trade-off between long-term carbon sequestration and immediate agronomic benefits.

### GHG emissions

Beyond carbon storage, biochar influences soil GHG fluxes, particularly carbon dioxide and nitrous oxide, which are directly linked to nitrogen cycling and soil respiration. Field experiments in Mediterranean agroecosystems generally report neutral or reduced carbon dioxide emissions following biochar application, indicating that biochar carbon is not rapidly mineralized [52, 53]. This finding supports the classification of biochar as a genuine carbon dioxide removal strategy rather than a temporary carbon storage mechanism. Biochar has demonstrated a consistent capacity to reduce nitrous oxide emissions in Mediterranean agricultural soils, with reported reductions ranging from 30 to 70% relative to unamended controls [28, 54]. The underlying mechanisms include adsorption of ammonium and nitrate ions, which reduces substrate availability for nitrification and denitrification, as well as improved soil aeration that limits anaerobic microsites where denitrification occurs [21, 22, 55]. Mediterranean studies highlight that nitrous oxide mitigation is particularly pronounced when biochar is combined with organic fertilizers or digestates, as biochar stabilizes labile carbon inputs that would otherwise stimulate rapid microbial activity and nitrous oxide production [54, 55]. These findings suggest that integrated nutrient management strategies enhance both the climate and crop health benefits of biochar.

In contrast to the generally positive effects on carbon dioxide and nitrous oxide, biochar effects on methane emissions in Mediterranean agroecosystems are more complex and potentially problematic (Table 2) [8, 21, 28, 34, 50, 52, 54, 56–65]. As illustrated in Fig. 1, these GHG dynamics reflect the balance between beneficial processes such as nitrous oxide mitigation and potential trade-offs, including methane emissions under high soil moisture conditions. Several long-term field studies report increased methane emissions following biochar application, particularly under warm and moist soil conditions typical of Mediterranean autumn and winter seasons [28, 34]. The primary mechanism driving methane emissions appears to be the formation of biologically induced anaerobic microsites within biochar pore structures when soils become water saturated. These microsites create localized oxygen-limited conditions that favor methanogenic microorganisms. In addition, biochar may influence methane emissions by altering microbial community composition and, under certain conditions, providing labile carbon substrates that stimulate methanogenic activity [28]. These microsites promote methanogenic activity, especially under conditions of high soil moisture following prolonged dry periods. Llovet et al. [34] demonstrated that methane emissions increased proportionally with biochar application rate, highlighting a trade-off between maximizing carbon sequestration and minimizing GHG emissions. Evidence from Mediterranean studies further indicates that methane emissions may increase with higher biochar application rates, suggesting a trade-off between maximizing carbon sequestration and minimizing GHG emissions. Optimizing application rates is therefore critical, with moderate rates (generally 10–30 Mg ha⁻^1^) representing a balance between agronomic benefits, carbon storage, and GHG mitigation under Mediterranean conditions [34]. This trade-off is particularly relevant for Mediterranean carbon farming strategies, as modest increases in methane emissions can offset a significant fraction of the climate benefits associated with carbon sequestration. Consequently, net climate assessments that exclude methane fluxes risk overestimating the mitigation potential of biochar, especially in fine-textured or poorly drained soils.

**Table 2:** Effects of biochar on soil properties and environmental processes in Mediterranean agroecosystems

**Category**	**Parameter**	**Effect of biochar**	**Quantification** (if available)	**Ref**
**Soil** **physical** **properties**	Soil porosity	↑ soil porosity	—	Alessandrino et al. [56]
	Pore size distribution	↑ medium and fine pores	—	Ibrahim et al. [57]
	Soil structure	↑ aeration; ↑ improved structure	—	EL-Sayed et al. [58]
	Water retention	↑ soil water content	—	De la Rosa et al. [59]
	Bulk density compaction	↓ soil compaction	—	Rombolà et al. [50]
	Hydraulic conductivity	↓ saturated hydraulic conductivity	—	Aguirre et al. [60]
**Soil chemical croperties**	SOC	↑ SOC stabilization	+ 39%	Bolan et al. [8]
	CEC	↑ CEC	—	Doulgeris et al. [61]
	Soil pH	↑ pH (moderate effect)	—	De la Rosa et al. [59]
	Nutrient leaching	↓ nutrient losses	—	Martos et al. [62]
	Phosphorus availability	↑ phosphorus availability	up to × 2	Doulgeris et al. [61]
**Soil biological properties**	Microbial activity	↑ microbial activity and diversity	—	Moreno et al. [63]
	FDA activity	↑ fluorescein diacetate activity	—	Moreno et al. [63]
	DHA activity	↑ dehydrogenase activity	—	Wojewódzki et al. [64]
	Urease activity	↑ urease activity	—	Moreno et al. [63]
	Phosphatase activity	± variable (biochar-dependent)	—	Wojewódzki et al. [64]
**Environment**	Carbon sequestration	↑ long-term carbon storage	—	Ribas et al. [28]
	CO_2_ emissions	± neutral	—	Castaldi et al. [52]
	N_2_O emissions	↓ emissions	− 30% to − 70%	Lagomarsino et al. [54]
	CH_4_ emissions	↑ emissions under wet conditions	rate-dependent	Llovet et al. [34]
	NH_3_ volatilization	↓ NH_3_ emissions	—	Chu et al. [21]
	Heavy metals mobility	↓ bioavailability	—	Lilli et al. [65]

↑ indicates increase; ↓ indicates decrease; ± indicates variability or context-dependent response

Abbreviations: CEC, cation exchange capacity; CH_4_, methane; CO_2_, carbon dioxide; DHA, dehydrogenase activity; FDA, fluorescein diacetate activity; N_2_O, nitrous oxide; NH_3_, ammonia; SOC, soil organic carbon

### Implications for climate-smart crop management

Soil health represents a critical mechanistic link between biochar-based carbon farming strategies and crop health outcomes in Mediterranean agroecosystems. While biochar application consistently modifies physical, chemical, and biological soil properties, these changes translate into agronomic benefits only when they alleviate crop-limiting constraints such as water scarcity, nutrient imbalance, or biological stress. This section synthesizes soil health mechanisms that are most relevant for explaining observed crop responses under Mediterranean conditions, as illustrated in Fig. 1.

Mediterranean agricultural soils frequently exhibit structural degradation, low aggregate stability, and reduced water-holding capacity because of intensive tillage, organic matter depletion, and prolonged dry periods [4]. As illustrated in Fig. 1, improvements in soil physical properties, including enhanced porosity, aggregate stability, and water retention, play a central role in mediating plant water availability and root development under Mediterranean conditions. Indeed, biochar application can improve soil physical properties by reducing bulk density, increasing porosity, and enhancing aggregate stability, thereby creating a more favorable root environment [20, 66, 67]. Several Mediterranean studies report that biochar increases medium and fine pore fractions, thereby improving soil water retention during dry periods without severely restricting aeration under optimal moisture conditions [59, 66]. These physical changes are directly linked to improved crop water status, delayed onset of drought stress, and enhanced water-use efficiency, particularly in coarse-textured or degraded soils. However, excessive biochar application or the use of fine-particle biochar may reduce saturated hydraulic conductivity and promote anaerobic microsite formation under wet conditions, potentially impairing root function and increasing methane emissions [28, 68]. This highlights the need for rate and texture optimization to balance water retention with adequate soil aeration.

Biochar influences soil chemical properties primarily through increased CEC, enhancing nutrient sorption, and buffering of nutrient losses. In Mediterranean soils, which are often calcareous and alkaline, biochar-induced pH changes are generally modest; nevertheless, aging processes can enhance surface functional groups and increase nutrient retention capacity over time [69]. Numerous Mediterranean studies demonstrate that biochar application increases CEC and reduces nutrient leaching, particularly for nitrogen and potassium, thereby improving fertilizer-use efficiency [62]. Enhanced nutrient buffering supports more stable nutrient supply to crops, reducing the risk of nutrient stress during critical growth stages. Figure 1 further highlights how biochar-induced changes in soil chemical properties, such as increased CEC and improved nutrient retention, contribute to more stable nutrient availability and reduced losses in Mediterranean agroecosystems. However, nutrient immobilization represents a potential short-term constraint, especially for nitrogen and phosphorus, immediately following biochar application [70, 71]. This effect is more pronounced when biochar is applied without complementary nutrient inputs and can temporarily limit crop growth, as observed in some Mediterranean fields and controlled environment studies [72, 73]. Phosphorus dynamics in biochar-amended Mediterranean soils remain particularly complex. While increased soil moisture and improved structure can enhance phosphorus availability [61, 74], biochar may also adsorb phosphate or promote precipitation with calcium, reducing plant availability in alkaline soils [75]. These contrasting effects help explain variable crop responses observed in phosphorus-sensitive species and highlight the importance of soil testing and amendment-specific management.

Biological communities in soil play a central role in mediating biochar effects on crop health. Biochar’s porous structure provides a physical habitat for microorganisms, protects them from desiccation during dry periods, and modifies nutrient availability in the rhizosphere [76]. In Mediterranean agroecosystems, biochar application has been shown to increase microbial diversity and shift community composition toward functionally beneficial groups, including plant growth–promoting rhizobacteria and arbuscular mycorrhizal fungi [77]. These biological changes contribute to enhanced nutrient acquisition, particularly phosphorus and micronutrients, and can improve crop tolerance to abiotic stresses such as drought and salinity [69]. Additionally, biochar-amended soils have demonstrated increased suppression of soil-borne pathogens, reducing disease incidence in vegetable cropping systems through modulation of soil microbiota [78].

However, biological responses to biochar are not universally beneficial. Under high soil moisture conditions, biochar may promote anaerobic microbial processes, shifting communities toward denitrification and methanogenesis, with negative implications for both crop root health and GHG emissions [28, 34]. These findings emphasize that moisture management is a critical determinant of whether biochar-induced biological changes enhance or impair crop performance. Although biochar frequently improves soil properties, these improvements do not always result in enhanced crop health or yield. Situations in which soil benefits fail to translate into agronomic gains include non-limiting soil conditions, excessive application rates, nutrient imbalances, or unfavorable interactions with specific crop rooting strategies [53, 71, 76]. For example, biochar-induced increases in water retention may compete with plant roots for moisture in sandy soils or reduce oxygen availability in fine-textured soils, negatively affecting sensitive crops such as peach trees (*Prunus persica*) [79, 80]. The role of biological processes in soil is also emphasized in Fig. 1, where biochar is shown to promote beneficial microbial communities and rhizosphere interactions that enhance nutrient cycling and plant resilience to environmental stress. These outcomes underline a central conclusion for Mediterranean carbon farming: soil health improvements must be evaluated in relation to crop-specific requirements and environmental context. Biochar is most effective when soil constraints limit crop performance, whereas blanket application in already well-functioning soils may provide limited or even negative returns.

From a crop health perspective, the climate mitigation potential of biochar must be evaluated alongside its agronomic effects. Biochar application can enhance crop resilience by improving water availability and nutrient retention, yet excessive application rates or poorly timed amendments may increase methane emissions without delivering proportional yield benefits [34, 76]. Evidence from Mediterranean systems suggests that moderate application rates (generally 10–30 Mg ha^−1^) optimize the balance between carbon sequestration, GHG mitigation, and crop performance [19, 53, 71]. Optimizing biochar use within Mediterranean carbon farming therefore requires careful consideration of soil texture, drainage, seasonal moisture patterns, and crop type. Strategies such as adjusting particle size, timing applications to avoid wet periods, and integrating biochar with complementary practices (e.g., cover cropping, organic fertilization, reduced tillage) may help minimize methane risks while preserving benefits for crop health and productivity [8, 30, 68].

## Crop Responses to Biochar in Mediterranean Systems

**Fig. 2:**
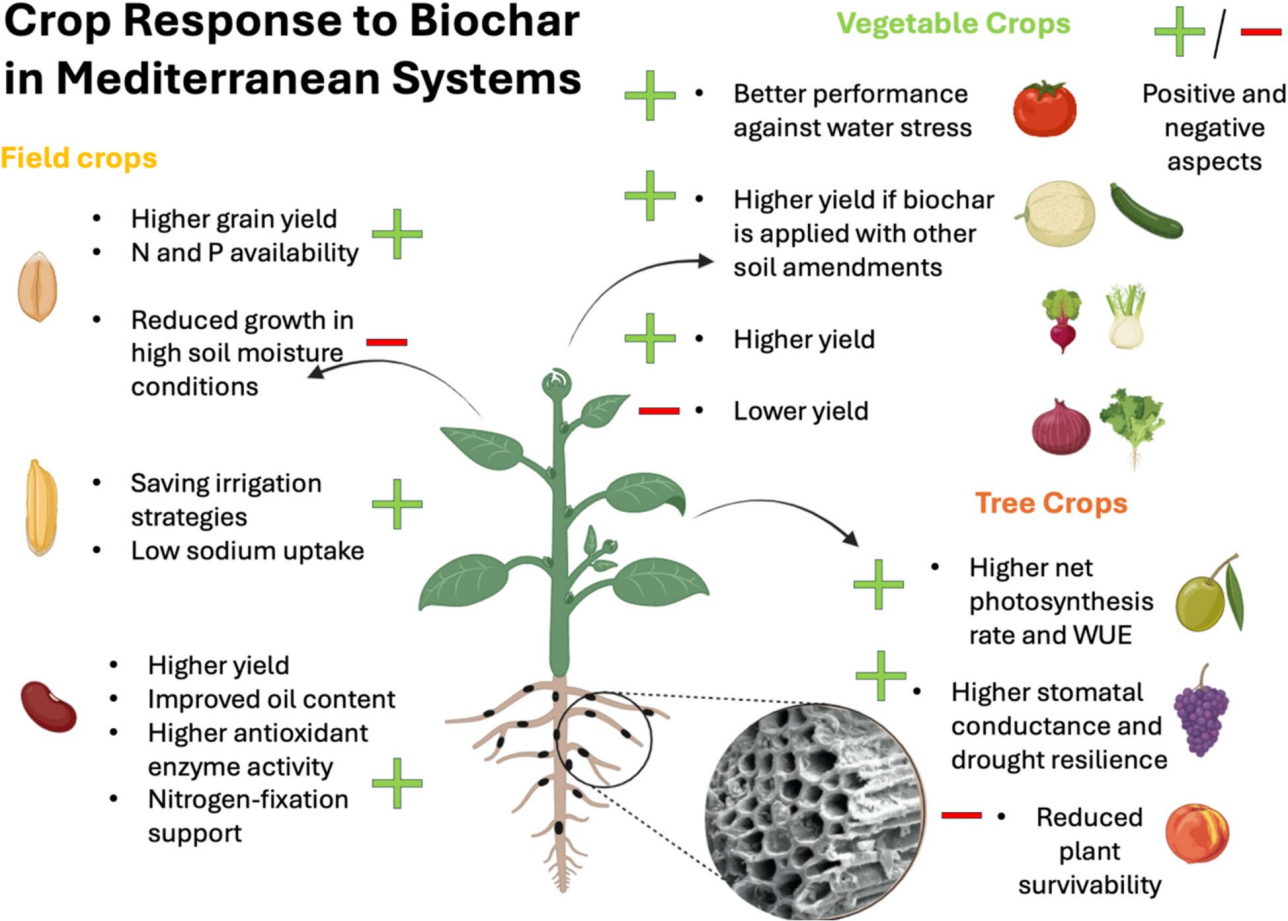
Crop responses to biochar application in Mediterranean agroecosystems under climate-smart carbon farming strategies. Green and red symbols report positive and negative plant responses, respectively. Abbreviations: N, nitrogen; P, phosphorus; WUE, water-use efficiency

Mediterranean cropping systems are increasingly exposed to concurrent abiotic stresses, including drought, heat extremes, salinity, and declining soil fertility, which directly compromise crop health, yield stability, and resource-use efficiency [7, 81]. Biochar has been proposed as a soil amendment capable of mitigating these constraints by modifying the physical, chemical, and biological soil conditions. However, crop responses to biochar application under Mediterranean conditions are highly variable and depend on the crop’s functional type, soil properties, biochar characteristics, application rate, and complementary management practices [53, 71, 76]. Crop responses to biochar application in Mediterranean agroecosystems are summarized in Fig. 2, which provides a synthetic overview of positive and negative responses across different crop groups, including field crops, vegetables, and perennial systems.

### Annual field crops: cereals and legumes

Among Mediterranean cereals, durum wheat (*Triticum durum* Desf.) has been the most extensively studied species. Field experiments conducted under Mediterranean conditions demonstrate that biochar application can increase wheat yield while enhancing soil carbon sequestration [44, 82]. Yield improvements are primarily attributed to enhanced soil water retention during grain filling and improved nitrogen and phosphorus availability [9, 52]. As illustrated in Fig. 2, field crops generally exhibit positive responses in terms of yield and nutrient availability under stress conditions, although negative effects on early growth may occur under high soil moisture or nutrient-limited conditions. Controlled environment and open field studies report reductions in early shoot growth, evapotranspiration, and projected shoot system area following biochar application, particularly when biochar is applied without complementary nutrient inputs [32, 72]. These negative responses are likely linked to temporary nutrient immobilization or reduced root-zone aeration under high soil moisture conditions. Collectively, these findings indicate that wheat benefits from biochar primarily under stress-prone environments rather than under optimal growing conditions (Fig. 2).

Rice (*Oryza sativa* L.) cultivated in Mediterranean regions shows more consistent positive responses when biochar is integrated with water-saving irrigation strategies. Studies combining biochar with sprinkler or deficit irrigation report increased yields, enhanced photosynthetic activity, and improved water-use efficiency compared with conventional flooded systems [79, 83, 84]. These results suggest that biochar can facilitate the transition toward less water-intensive rice production systems in Mediterranean climates, providing that soil moisture is carefully managed to limit anaerobic microsite formation and methane emissions [28]. Maize (*Zea mays* L.) responses to biochar are strongly context-dependent. In saline Mediterranean soils, biochar application mitigates salt stress by reducing sodium uptake, maintaining potassium availability, and enhancing photosynthetic performance, resulting in improved agronomic parameters and biomass accumulation [85, 86].

Conversely, medium-term field trials conducted under non-stress conditions report negligible yield responses despite measurable improvements in soil chemical properties and CEC [60]. This divergence highlights that soil improvement does not necessarily translate into yield gains unless soil constraints are a primary limiting factor for crop growth. Leguminous crops generally exhibit positive responses to biochar application under Mediterranean conditions. Fenugreek (*Trigonella foenum-graecum* L.) shows increased seed yield, mineral composition, and oil content following biochar amendment [87]. Faba bean (*Vicia faba* L.) and cowpea (*Vigna unguiculata* [L.] Walp.) demonstrate enhanced chlorophyll content, antioxidant enzyme activity, nutrient uptake, and yield under saline or water-deficit conditions [85, 88, 89]. These responses are likely driven by improved phosphorus availability and enhanced rhizosphere conditions that support biological nitrogen fixation, suggesting legumes are particularly suitable candidates for biochar-based interventions in Mediterranean crop rotations.

### Vegetables

Vegetable cropping systems represent high-value agricultural contexts in which improvements in crop health, quality, and stress resilience can offset the economic costs of biochar application. Among solanaceous crops, tomato (*Solanum lycopersicum* L.) consistently responds positively to biochar under Mediterranean conditions. Biochar-amended soils promote vegetative growth, increase fruit number and fruit weight, and enhance overall productivity, particularly during summer periods characterized by water stress [36, 90, 91]. These benefits are primarily attributed to improved soil water retention, reduced nutrient leaching, and stimulation of beneficial rhizosphere microbial communities. In particular, the coexistence of positive and negative responses illustrated in Fig. 2 highlights the importance of amendment-specific and crop-specific management strategies. Pepper (*Capsicum annuum* L. subsp. *melo*) also shows favorable responses to biochar application, especially when biochar is combined with organic amendments or biostimulants. Field studies report improved nutrient uptake, reduced phytoavailability of toxic elements in contaminated soils, and yield increases exceeding 30% compared with control treatments [77, 92]. These results highlight the importance of integrating biochar within broader soil fertility management strategies rather than applying it as a standalone amendment.

Responses among cucurbit crops are more variable (Fig. 2). Melon (*Cucumis melo* L.) exhibits yield increases when biochar is combined with anaerobic digestate, whereas zucchini (*Cucurbita pepo* L. var. *cylindrica*) shows improvements in chlorophyll content, germination rate, and vegetative growth following biochar amendment [77, 85, 93]. In contrast, leafy vegetables display the greatest variability. Biochar combined with specific compost formulations show increased yields of fennel (*Foeniculum vulgare* Mill*.*) and rape (*Brassica napus* L.), whereas there were reduced yields of lettuce (*Lactuca sativa* L.) and onion (*Allium cepa* L.) in volcanic Mediterranean soils [33]. Negative responses were associated with nutrient imbalances and the presence of phytotoxic compounds, underscoring the need for crop-specific and amendment-specific optimization (Table 3) [32, 33, 36, 59, 77, 80, 83, 84, 87–90, 93–96].

**Table 3:** Summary of the biochar effects on different species under Mediterranean agrienvironmental conditions

Botanical family	Species	Response type	Effect	Ref
Apiaceae	*Allium cepa*	Growth	↓ growth	Iacomino et al. [33]
	*Foeniculum vulgare*		↑ growth	
Asteraceae	*Lactuca sativa*	Growth, stress physiology, quality	↓ growth	Seleiman et al. [94]
	*Helianthus annuus*		↓ drought stress, ↑ oil quality	
Brassicaceae	*Brassica napus*	Growth	↑ growth	Iacomino et al. [33]
Cucurbitaceae	*Cucumis melo* and *cucurbita pepo*	Yield, growth, physiology	↑ germination, ↑ yield, ↑ chlorophyll content, ↑ root and shoot length	Ortiz-Liébana et al. [77], Farid et al. [93]
Fabaceae	*Vicia faba*	Physiology, yield, nutrition	↑ chlorophyll content, ↑ enzyme activity	El Nahhas et al. [88]
	*Trigonella foenum-graecum*		↑ yield, ↑ mineral composition	Qaisi et al. [87]
	*Vigna unguiculata*		↑ yield	El-Hassanin et al. [89]
Oleaceae	*Olea europaea*	Physiology, water relations	↑ soil moisture, ↑ photosynthesis, ↑ WUE, ↑ ETRmax	De la Rosa et al. [59]
Poaceae	*Oryza sativa*	Yield, water use, physiology, growth	↑ yield, ↑ WUE, ↑ chlorophyll, ↑ carotenoids, ↑ stomatal conductance, ↓ evapotranspiration, ↓ shoot growth	Rato-Nunes et al. [83], Elshayb et al. [84]
	*Triticum durum*			Hafez et al. [95], Latini et al. [32]
Rosaceae	*Prunus persica*	Root physiology	± root physiology, ↓ survivability	Sorrenti et al. [80]
Solanaceae	*Capsicum annuum*	Nutrition, stress, yield, growth	↑ nutrient uptake, ↓ toxic elements uptake, ↑ yield, ↑ vegetative growth, ↑ fruit number and weight, ↑ water retention	Ortiz-Liébana et al. [77]
	*Solanum lycopersicum*			Graber et al. [36], Velli et al. [90]
Vitaceae	*Vitis vinifera*	Stress physiology	↑ performance under stress, ↑ drought resilience	Baronti et al. [96]

↑ indicates increase; ↓ indicates decrease; ± indicates variable or context-dependent response

Abbreviations: ETR, electron transport rate; WUE, water-use efficiency

### Tree crops and vineyards

Perennial cropping systems provide a particularly suitable context for biochar application due to their long production cycles, which allow benefits to be accumulated over time. As shown in Fig. 2, perennial crops such as olive and grapevine exhibit the most consistent positive responses, particularly in terms of improved water-use efficiency, photosynthetic performance, and drought resilience. Olive (*Olea europaea* L.) orchards show strong positive responses to biochar under Mediterranean conditions. Biochar derived from olive residues increases soil moisture availability, net photosynthetic rate, water-use efficiency, and fruit yield, even under controlled deficit irrigation regimes [59, 85, 97]. These effects are especially relevant for super-intensive olive systems, where high planting density and limited water availability impose chronic stress on trees. Grapevines (*Vitis vinifera* L.) represent one of the most compelling cases for long-term biochar benefits. Field experiments demonstrate that a single biochar application can improve vine water status, stomatal conductance, and drought resilience for more than a decade [96, 98]. Such persistent effects substantially enhance the cost-effectiveness of biochar in Mediterranean viticulture and support its integration into climate-smart vineyard management. However, not all perennial crops respond favorably. Figure 2 indicates that not all perennial species respond positively, with some cases of reduced plant survivability, emphasizing the need for species-specific evaluation. Peach (*P. persica* [L.] Batsch) trees exhibit reduced survivability following biochar application, despite normal yield and fruit weight among surviving plants [80]. These negative effects are likely linked to biochar-induced alterations in root-zone water dynamics or physical interference with fine root development, highlighting the need for species-specific evaluation prior to large-scale adoption.

### Crop-type sensitivity to biochar in Mediterranean agriculture

Across Mediterranean agroecosystems, biochar improves crop health most consistently under conditions of environmental stress, particularly drought, salinity, and soil degradation [19, 47, 76]. Perennial crops and legumes emerge as the most responsive functional groups, while cereals and leafy vegetables exhibit more variable outcomes. Moderate application rates (typically 10–30 Mg ha⁻^1^) maximize agronomic benefits while minimizing risks associated with nutrient immobilization and anaerobic microsite formation [34, 70, 71]. These findings indicate that biochar should be deployed selectively within Mediterranean agriculture, targeting systems where soil constraints limit crop performance rather than applied uniformly across all cropping systems.

## Final remarks and future challenges

Mediterranean agriculture faces increasing pressure from climate change–driven stresses, including drought, SOM depletion, and declining yield stability. Within this context, carbon farming strategies aim to integrate climate mitigation with sustained crop health. This review shows that biochar represents a distinctive but context-dependent component of Mediterranean carbon farming, offering permanent carbon sequestration while influencing soil processes that regulate crop performance. Biochar application consistently increases SOC stocks through the addition of highly recalcitrant carbon forms that remain stable under Mediterranean climatic conditions. GHG responses, however, are not uniform. Biochar generally reduces nitrous oxide emissions (often by 30%–70%) and does not increase carbon dioxide emissions, supporting its mitigation potential. In contrast, methane emissions may increase under warm and moist conditions due to anaerobic microsite formation, potentially offsetting climate benefits if applications are not carefully managed. Comprehensive GHG accounting is therefore essential when evaluating biochar-based carbon farming. From a crop health perspective, biochar improves crop performance primarily when it alleviates limiting soil constraints. The most consistent benefits are observed in degraded or coarse-textured soils, under water-limited or saline conditions, and in perennial cropping systems. Perennial crops such as olive and grapevine show long-lasting improvements in water status, physiological performance, and yield stability, while legumes generally respond positively due to enhanced nutrient availability and rhizosphere functioning. In contrast, cereal crops and leafy vegetables display more variable responses, including neutral or negative effects when soil conditions are already non-limiting or when nutrient imbalances occur. Future research should prioritize long-term field experiments in Mediterranean soils to validate crop yield persistence, carbon stability, and net GHG balances. Particular attention should be given to methane mitigation strategies and to underexplored cropping systems, especially legumes and diversified rotations. Translating this evidence into decision-support tools will be critical for guiding farmers and policymakers toward effective and context-specific biochar deployment.

Beyond agronomic and environmental performance, the economic viability of biochar application represents a critical factor for its adoption in Mediterranean agriculture. The overall cost of biochar systems includes several components, notably feedstock collection and preparation, pyrolysis processing, transportation, and field application. These costs can be substantial, particularly in Mediterranean regions characterized by fragmented landownership and limited access to processing infrastructure. However, potential economic benefits may partially offset these costs, including improved crop productivity, enhanced nutrient-use efficiency, and reduced input requirements over time. In addition, the long-term carbon sequestration potential of biochar opens opportunities for participation in carbon credit markets, which could provide financial incentives for farmers adopting biochar-based practices. Despite these opportunities, uncertainties in carbon pricing and the lack of standardized policy frameworks across Mediterranean countries remain significant barriers. Future research should therefore integrate agronomic, environmental, and economic assessments to support the development of cost-effective and scalable biochar strategies tailored to Mediterranean agroecosystems.

## Data Availability

The datasets generated during and/or analyzed during the current study are available from the corresponding author on reasonable request.

## References

[1] Batibeniz F, Ashfaq M, Önol B, Turuncoglu UU, Mehmood S, Evans KJ. Identification of major moisture sources across the Mediterranean Basin. Clim Dyn. 2020;54:4109–27. 10.1007/s00382-020-05224-3.

[2] Lionello P, Scarascia L. The relation between climate change in the Mediterranean region and global warming. Reg Environ Change. 2018;18:1481–93. 10.1007/s10113-018-1290-1.

[3] Magory Cohen T, Hauber ME, Akriotis T, Crochet PA, Karris G, Kirschel AN, et al. Accelerated avian invasion into the Mediterranean region endangers biodiversity and mandates international collaboration. J Appl Ecol. 2022;59(6):1440–55. 10.1111/1365-2664.14150.

[4] Mohammed S, Jouhra A, Enaruvbe GO, Bashir B, Barakat M, Alsilibe F, et al. Performance evaluation of machine learning algorithms to assess soil erosion in Mediterranean farmland: A case-study in Syria. Land Degrad Dev. 2023;34(10):2896–911. 10.1002/ldr.4655.

[5] Busico G, Grilli E, Carvalho SC, Mastrocicco M, Castaldi S. Assessing soil erosion susceptibility for past and future scenarios in semiarid Mediterranean agroecosystems. Sustainability. 2023;15(17):12992. 10.3390/su151712992.

[6] Doussin JF. The Mediterranean atmosphere under anthropogenic pressures. In: Dulac F, Sauvage S, Hamonou E, editors. Atmospheric chemistry in the Mediterranean region, Vol. 1 - background information and pollutant distribution. Cham: Springer International Publishing; 2023. p. 77–98. 10.1007/978-3-031-12741-0_4.

[7] Zittis G, Almazroui M, Alpert P, Ciais P, Cramer W, Dahdal Y, et al. Climate change and weather extremes in the Eastern Mediterranean and Middle East. Rev Geophys. 2022;60(3):e2021RG000762. 10.1029/2021RG000762.

[8] Bolan N, Hoang SA, Beiyuan J, Gupta S, Hou D, Karakoti A, et al. Multifunctional applications of biochar beyond carbon storage. Inter Mater Rev. 2022;67:150–200. 10.1080/09506608.2021.1922047.

[9] Ibrahim SSS, Abd-Elhay YB, Elkoly MM, Suliman AE, Mohamed BA. Catalyzed biochar from date palm waste for ammonium removal: potential application in poultry farms for ammonia mitigation. Discov Sustain. 2025;6(1):652. 10.1007/s43621-025-00817-6.

[10] Duval ME, Galantini JA, Capurro JE, Martinez JM. Winter cover crops in soybean monoculture: effects on soil organic carbon and its fractions. Soil Tillage Res. 2016;161:95–105. 10.1016/j.still.2016.04.006.

[11] Schmidt H, Kammann C, Hagemann N, Leifeld J, Bucheli TD, Sánchez Monedero MA, et al. Biochar in agriculture – a systematic review of 26 global meta‐analyses. GCB Bioenergy. 2021;13:1708–30. 10.1111/gcbb.12889.

[12] Premchand P, Demichelis F, Chiaramonti D, Bensaid S, Fino D. Biochar production from slow pyrolysis of biomass under CO_2_ atmosphere: A review on the effect of CO_2_ medium on biochar production, characterisation, and environmental applications. J Environ Chem Eng. 2023;11:110009. 10.1016/j.jece.2023.110009.

[13] Al-Rumaihi A, Shahbaz M, Mckay G, Mackey H, Al-Ansari T. A review of pyrolysis technologies and feedstock: A blending approach for plastic and biomass towards optimum biochar yield. Renew Sustain Energy Rev. 2022;167:112715. 10.1016/j.rser.2022.112715.

[14] Li Z, Shi X. Towards sustainable industrial application of carbon-negative concrete: synergistic carbon-capture by concrete washout water and biochar. Mater Lett. 2023;342:134368. 10.1016/j.matlet.2023.134368.

[15] Safarian S. Performance analysis of sustainable technologies for biochar production: A comprehensive review. Energy Rep. 2023;9:4574–93. 10.1016/j.egyr.2023.03.111.

[16] Keith A, Singh B, Singh BP. Interactive priming of biochar and labile organic matter mineralization in a smectite-rich soil. Environ Sci Technol. 2011;45:9611–8. 10.1021/es202186j. 21950729 10.1021/es202186j

[17] Singh H, Northup BK, Rice CW, Prasad PVV. Biochar applications influence soil physical and chemical properties, microbial diversity, and crop productivity: a meta-analysis. Biochar. 2022;4:8. 10.1007/s42773-022-00138-1.

[18] Ngoc-Dan Cao T, Mukhtar H, Yu C-P, Bui X-T, Pan S-Y. Agricultural waste-derived biochar in microbial fuel cells towards a carbon-negative circular economy. Renew Sustain Energy Rev. 2022;170:112965. 10.1016/j.rser.2022.112965.

[19] Nogués I, Mazzurco Miritana V, Passatore L, Zacchini M, Peruzzi E, et al. Biochar soil amendment as carbon farming practice in a Mediterranean environment. Geoderma Reg. 2023;33:e00634. 10.1016/j.geodrs.2023.e00634.

[20] Blanco-Canqui H. Biochar and soil physical properties. Soil Sci Soc Am J. 2017;81:687–711. 10.2136/sssaj2017.01.0017.

[21] Chu C, Dai S, Meng L, Cai Z, Zhang J, Müller C. Biochar application can mitigate NH3 volatilization in acidic forest and upland soils but stimulates gaseous N losses in flooded acidic paddy soil. Sci Total Environ. 2023;864:161099. 10.1016/j.scitotenv.2022.161099. 36572316 10.1016/j.scitotenv.2022.161099

[22] Hale L, Hendratna A, Scott N, Gao S. Biochar enhancement of nitrification processes varies with soil conditions. Sci Total Environ. 2023;887:164146. 10.1016/j.scitotenv.2023.164146. 37182767 10.1016/j.scitotenv.2023.164146

[23] O’Neil CM, Nash J, Tiemann LK, Miesel JR. Mycorrhizal symbioses enhance competitive weed growth in biochar and nutrient-amended. Soils Front Agron. 2021;3:731184. 10.3389/fagro.2021.731184.

[24] Xu M, Ma J, Zhang X-H, Yang G, Long L-L, Chen C, et al. Biochar-bacteria partnership based on microbially induced calcite precipitation improves Cd immobilization and soil function. Biochar. 2023;5:20. 10.1007/s42773-023-00222-0.

[25] Prommer J, Wanek W, Hofhansl F, Trojan D, Offre P, Urich T, et al. Biochar decelerates soil organic nitrogen cycling but stimulates soil nitrification in a temperate arable field trial. PLoS ONE. 2014;9:e86388. 10.1371/journal.pone.0086388. 24497947 10.1371/journal.pone.0086388 PMC3907405

[26] Murtaza G, Ahmed Z, Eldin SM, Ali B, Bawazeer S, Usman M, et al. Biochar-Soil-Plant interactions: A cross talk for sustainable agriculture under changing climate. Front Environ Sci. 2023;11:1059449. 10.3389/fenvs.2023.1059449.

[27] Giannetta B, Plaza C, Cassetta M, Mariotto G, Benavente-Ferraces I, et al. The effects of biochar on soil organic matter pools are not influenced by climate change. J Environ Manage. 2023;341:118092. 10.1016/j.jenvman.2023.118092. 37167698 10.1016/j.jenvman.2023.118092

[28] Ribas A, Mattana S, Llurba R, Debouk H, Sebastià MT, Domene X. Biochar application and summer temperatures reduce N_2_O and enhance CH_4_ emissions in a Mediterranean agroecosystem: role of biologically-induced anoxic microsites. Sci Total Environ. 2019;685:1075–86. 10.1016/j.scitotenv.2019.06.277. 31390698 10.1016/j.scitotenv.2019.06.277

[29] Wang J, Wang S. Preparation, modification and environmental application of biochar: a review. J Clean Prod. 2019;227:1002–22. 10.1016/j.jclepro.2019.04.282.

[30] Zabaniotou A, Stamou K. Balancing waste and nutrient flows between urban agglomerations and rural ecosystems: biochar for improving crop growth and urban air quality in the Mediterranean region. Atmosphere (Basel). 2020;11(5):539. 10.3390/atmos11050539.

[31] Dewees SL, D’Antonio CM, Molinari N. Determining potential drivers of vegetation change in a Mediterranean environment. Ecosphere. 2022;13(12):e4313. 10.1002/ecs2.4313.

[32] Latini A, Fiorani F, Galeffi P, Cantale C, Bevivino A, Jablonowski ND. Phenotyping of different Italian durum wheat varieties in early growth stage with the addition of pure or digestate-activated biochars. Front Plant Sci. 2021;12:782072. 10.3389/fpls.2021.782072. 34987533 10.3389/fpls.2021.782072 PMC8721205

[33] Iacomino G, Sarker TC, Ippolito F, Bonanomi G, Vinale F, Staropoli A, et al. Biochar and compost application either alone or in combination affects vegetable yield in a volcanic mediterranean soil. Agronomy. 2022;12:1996. 10.3390/agronomy12091996.

[34] Llovet A, Mattana S, Chin-Pampillo J, Gascó G, Sánchez S, Mondini C, et al. Long-term effects of gasification biochar application on soil functions in a Mediterranean agroecosystem: Higher addition rates sequester more carbon but pose a risk to soil faunal communities. Sci Total Environ. 2021;801:149580. 10.1016/j.scitotenv.2021.149580. 34411789 10.1016/j.scitotenv.2021.149580

[35] Dumbrell NP, Kragt ME, Gibson FL. What carbon farming activities are farmers likely to adopt? A best–worst scaling survey. Land Use Policy. 2016;54:29–37. 10.1016/j.landusepol.2016.02.002.

[36] Graber ER, Meller Harel Y, Kolton M, Cytryn E, Silber A, Rav David D, et al. Biochar impact on development and productivity of pepper and tomato grown in fertigated soilless media. Plant Soil. 2010;337:481–96. 10.1007/s11104-010-0544-6.

[37] Angın D. Effect of pyrolysis temperature and heating rate on biochar obtained from pyrolysis of safflower seed press cake. Bioresour Technol. 2013;128:593–7. 10.1016/j.biortech.2012.10.150. 23211485 10.1016/j.biortech.2012.10.150

[38] Agrafioti E, Bouras G, Kalderis D, Diamadopoulos E. Biochar production by sewage sludge pyrolysis. J Anal Appl Pyrolysis. 2013;101:72–8. 10.1016/j.jaap.2013.02.010.

[39] Pallarés J, González-Cencerrado A, Arauzo I. Production and characterization of activated carbon from barley straw by physical activation with carbon dioxide and steam. Biomass Bioenerg. 2018;115:64–73. 10.1016/j.biombioe.2018.04.015.

[40] Fellet G, Marchiol L, Delle Vedove G, Peressotti A. Application of biochar on mine tailings: effects and perspectives for land reclamation. Chemosphere. 2011;83(9):1262–7. 10.1016/j.chemosphere.2011.03.053. 21501855 10.1016/j.chemosphere.2011.03.053

[41] Mendez A, Gomez A, Paz-Ferreiro J, Gasco G. Effects of sewage sludge biochar on plant metal availability after application to a Mediterranean soil. Chemosphere. 2012;89(11):1354–9. 10.1016/j.chemosphere.2012.05.092. 22732302 10.1016/j.chemosphere.2012.05.092

[42] Özçimen D, Ersoy-Meriçboyu A. Characterization of biochar and bio-oil samples obtained from carbonization of various biomass materials. Renew Energy. 2010;35(6):1319–24. 10.1016/j.renene.2009.11.042.

[43] Agrafioti E, Kalderis D, Diamadopoulos E. Arsenic and chromium removal from water using biochars derived from rice husk, organic solid wastes and sewage sludge. J Environ Manage. 2014;133:309–14. 10.1016/j.jenvman.2013.12.007. 24412594 10.1016/j.jenvman.2013.12.007

[44] Vaccari FP, Baronti S, Lugato E, Genesio L, Castaldi S, Fornasier F, et al. Biochar as a strategy to sequester carbon and increase yield in durum wheat. Eur J Agron. 2011;34(4):231–8. 10.1016/j.eja.2011.01.006.

[45] Kolton M, Meller Harel Y, Pasternak Z, Graber ER, Elad Y, Cytryn E. Impact of biochar application to soil on the root-associated bacterial community structure of fully developed greenhouse pepper plants. Appl Environ Microbiol. 2011;77(14):4924–30. 10.1128/AEM.00148-11. 21622786 10.1128/AEM.00148-11 PMC3147372

[46] van Eck N, Waltman L. Software survey: VOSviewer, a computer program for bibliometric mapping. Scientometrics. 2010;84(2):523–38. 10.1007/s11192-009-0146-3. 20585380 10.1007/s11192-009-0146-3 PMC2883932

[47] Rani M, Abideen Z, Munir N, Hasnain M, Mehdizadeh M, Qasim M, et al. Enhancing soil fertility, nutrient recovery and carbon sequestration: the role of biochar, composted biochar, and biochar-compost mixtures in sustainable agriculture. J Trace Elem Min. 2026;15:100276. 10.1016/j.jtemin.2025.100276.

[48] Li H, Azzi ES, Sundberg C, Karltun E, Cederlund H. Can inert pool models improve predictions of biochar long-term persistence in soils? Geoderma. 2024;452:117093. 10.1016/j.geoderma.2024.117093.

[49] Wang L, Deng J, Yang X, Hou R, Hou D. Role of biochar toward carbon neutrality. Carbon Res. 2023;2:2. 10.1007/s44246-023-00035-7.

[50] Rombolà AG, Torri C, Vassura I, Venturini E, Reggiani R, Fabbri D. Effect of biochar amendment on organic matter and dissolved organic matter composition of agricultural soils from a two-year field experiment. Sci Total Environ. 2022;812:151422. 10.1016/j.scitotenv.2021.151422. 34742976 10.1016/j.scitotenv.2021.151422

[51] Sounni KA, Camps-Arbestain M, Kaal J, Tighe CJ, Titirici MM, Siavalas G. Assessment and integration of different methodologies for the characterisation of carbon aromaticity and structure in biochar. Int J Coal Geol. 2026;313:104925. 10.1016/j.coal.2025.104925.

[52] Castaldi S, Riondino M, Baronti S, Esposito FR, Marzaioli R, Rutigliano FA, et al. Impact of biochar application to a Mediterranean wheat crop on soil microbial activity and greenhouse gas fluxes. Chemosphere. 2011;85:1464–71. 10.1016/j.chemosphere.2011.08.031. 21944041 10.1016/j.chemosphere.2011.08.031

[53] Lan P, Chen Q, Wu M, Oleszczuk P, Pan B. Oligotrophy biochar stimulates the generation of salicylic acid from soybean roots by increasing nutrient and oxidative stress. Environ Technol Innov. 2025;38:104083. 10.1016/j.eti.2025.104083.

[54] Lagomarsino A, Valagussa M, Scotti C, Borrelli L, Becagli C, Tosca A. Mitigation of GHG emissions from soils fertilized with livestock chain residues. Agronomy. 2022;12:1593. 10.3390/agronomy12071593.

[55] Yang M, Guo J, Yan K, Huang M, Zhang H, Feng H. Biochar addition reduces soil greenhouse gas fluxes in terrestrial ecosystems. J Clean Prod. 2026;540:147521. 10.1016/j.jclepro.2026.147521.

[56] Alessandrino L, Pavlakis C, Colombani N, Mastrocicco M, Aschonitis V. Effects of graphene on soil water-retention curve, van Genuchten parameters, and soil pore size distribution—a comparison with traditional soil conditioners. Water. 2023;15:1297. 10.3390/w15071297.

[57] Ibrahim M, Mahmoud E, Gad L, Khader A. Effects of biochar and phosphorus fertilizer rates on soil physical properties and wheat yield on clay textured soil in middle Nile Delta of Egypt. Commun Soil Sci Plant Anal. 2019;50:2756–66. 10.1080/00103624.2019.1679162.

[58] EL-Sayed MM, Mahdy AY, Gebreel M, Abdeen SA. Effectiveness of biochar, organic matter and Mycorrhiza to improve soil hydrophysical properties and water relations of soybean under arid soil conditions. Eurasian Soil Sci. 2023;56:1055–66. 10.1134/S1064229323600276.

[59] De la Rosa JM, Campos P, Diaz-Espejo A. Soil biochar application: assessment of the effects on soil water properties, plant physiological status, and yield of super-intensive olive groves under controlled irrigation conditions. Agronomy. 2022;12:2321. 10.3390/agronomy12102321.

[60] Aguirre JL, González-Egido S, González-Lucas M, González-Pernas FM. Medium-term effects and economic analysis of biochar application in three Mediterranean crops. Energies. 2023;16:4131. 10.3390/en16104131.

[61] Doulgeris C, Kypritidou Z, Kinigopoulou V, Hatzigiannakis E. Simulation of potassium availability in the application of biochar in agricultural soil. Agronomy. 2023;13:784. 10.3390/agronomy13030784.

[62] Martos S, Mattana S, Ribas A, Albanell E, Domene X. Biochar application as a win-win strategy to mitigate soil nitrate pollution without compromising crop yields: a case study in a Mediterranean calcareous soil. J Soils Sediments. 2020;20:220–33. 10.1007/s11368-019-02400-9.

[63] Moreno JL, Bastida F, Díaz-López M, Li Y, Zhou Y, López-Mondéjar R, et al. Response of soil chemical properties, enzyme activities and microbial communities to biochar application and climate change in a Mediterranean agroecosystem. Geoderma. 2022;407:115536. 10.1016/j.geoderma.2021.115536.

[64] Wojewódzki P, Lemanowicz J, Debska B, Haddad SA. Soil enzyme activity response under the amendment of different types of biochar. Agronomy. 2022;12:569. 10.3390/agronomy12030569.

[65] Lilli MA, Paranychianakis NV, Lionoudakis K, Kritikaki A, Voutsadaki S, Saru ML, et al. The impact of sewage-sludge- and olive-mill-waste-derived biochar amendments to tomato cultivation. Sustainability. 2023;15:3879. 10.3390/su15053879.

[66] Guo J, Zhou H, Jia L, Wang Y, Fan M. Effects of biochar from different pyrolysis temperatures on soil physical properties and hydraulic characteristics in potato farmland of arid and semi-arid regions. Agric Water Manag. 2025;313:109483. 10.1016/j.agwat.2025.109483.

[67] Baiamonte G, Crescimanno G, Parrino F, De Pasquale C. Effect of biochar on the physical and structural properties of a sandy soil. CATENA. 2019;175:294–303. 10.1016/j.catena.2018.12.019.

[68] Bezzalla A, Bennadji MEA, Zidane L, Neffar S, Chenchouni H. A review on the impact of biochar applications on soil health and fertility, plant growth, and food security: advancing agricultural sustainability. Biomass Bioenerg. 2026;209:108960. 10.1016/j.biombioe.2026.108960.

[69] Safahani Langeroodi A, Tedeschi P, Allevato E, Stazi SR, Aadil RM, et al. Agronomic response of sunflower subjected to biochar and arbuscular mycorrhizal fungi application under drought conditions. Ital J Agron. 2022;17(3):2086. 10.4081/ija.2022.2086.

[70] Luo W, Liu Q, Cao H, Ren S, Zhang M, Liu S. Enrichment of nitrogen and phosphorus by navel orange peel–derived biochars coated with different metal (hydro)oxides for the subsequent immobilization of Cd in soil. J Environ Manage. 2025;392:126675. 10.1016/j.jenvman.2025.126675. 40690869 10.1016/j.jenvman.2025.126675

[71] Brtnicky M, Datta R, Holatko J, Bielska L, Gusiatin ZM, Kucerik J. A critical review of the possible adverse effects of biochar in the soil environment. Sci Total Environ. 2021;796:148756. 10.1016/j.scitotenv.2021.148756. 34273836 10.1016/j.scitotenv.2021.148756

[72] Lustosa Filho JF, Lima BC, Costa JDL, Santos MGBD, Costa CR, Oliveira SSD, et al. Oxalic acid enhances the performance of potassium-enriched biochar-based fertilizers. Biomass Bioenerg. 2026;208:108912. 10.1016/j.biombioe.2025.108912.

[73] Latini A, Giagnacovo G, Campiotti CA, Bibbiani C, Mariani S. A narrative review of the facts and perspectives on agricultural fertilization in Europe, with a focus on Italy. Horticulturae. 2021;7:158. 10.3390/horticulturae7060158.

[74] Frene JP, Kasera N, Jaisi DP, Sapkota S, O’Connell DW, Higgins S, et al. Enhancing soil health and phosphorus use efficiency with modified biochar amendment. Sci Total Environ. 2025;1004:180794. 10.1016/j.scitotenv.2025.180794. 41166967 10.1016/j.scitotenv.2025.180794

[75] Eltohamy KM, Milham PJ, Gouda M, Menezes-Blackburn D, Khan S, Liu B, et al. Size and composition of colloidal phosphorus across agricultural soils amended with biochar, manure and biogas slurry. Carbon Res. 2023;2:16. 10.1007/s44246-023-00048-2.

[76] Subedi R, Bertora C, Zavattaro L, Grignani C. Crop response to soils amended with biochar: expected benefits and unintended risks. Ital J Agron. 2017;12(2):794. 10.4081/ija.2017.794.

[77] Ortiz-Liébana N, Zotti M, Barquero M, González-Andrés F. Biochar + AD exerts a biostimulant effect in the yield of horticultural crops and improves bacterial biodiversity and species richness in the rhizosphere. Sci Hortic. 2023;321:112277. 10.1016/j.scienta.2023.112277.

[78] Bonanomi G, Alioto D, Minutolo M, Marra R, Cesarano G, Vinale F. Organic amendments modulate soil microbiota and reduce virus disease incidence in the TSWV-tomato pathosystem. Pathogens. 2020;9:379. 10.3390/pathogens9050379. 32423086 10.3390/pathogens9050379 PMC7281679

[79] Zhou L, Tao H, Qiliang Y, Feng H, Siddique KHM, Jin T. Biochar particle size shapes soil water–oxygen conditions and delays senescence in sweet corn under mulched drip irrigation. Soil Tillage Res. 2026;258:107049. 10.1016/j.still.2025.107049.

[80] Sorrenti G, Muzzi E, Toselli M. Root growth dynamic and plant performance of nectarine trees amended with biochar and compost. Sci Hortic. 2019;257:108710. 10.1016/j.scienta.2019.108710.

[81] Garrote L, Sordo-Ward A, Bianucci P, Martin-Carrasco F, Iglesias A. From global climate models to local water stress: A framework for estimating future water availability in the Mediterranean. J Hydrol Reg Stud. 2025;62:102960. 10.1016/j.ejrh.2025.102960.

[82] Chagas JKM, Figueiredo CCD. Combining meta-analysis and local assessment: An in-depth approach on biochar use towards soil carbon sequestration. Next Sustainability. 2026;7:100243. 10.1016/j.nxsust.2025.100243.

[83] Rato-Nunes JM, Martín-Franco C, Peña D, Terrón-Sánchez J, Vicente LA, Fernández-Rodríguez D, et al. Combined use of biochar and sprinkler irrigation may enhance rice productivity in water-stressed regions. Ann Agric Sci. 2023;68:48–59. 10.1016/j.aoas.2023.05.002.

[84] Elshayb OM, Nada AM, Sadek AH, Ismail SH, Shami A, Alharbi BM, et al. The integrative effects of biochar and ZnO nanoparticles for enhancing rice productivity and water use efficiency under irrigation deficit conditions. Plants. 2022;11:1416. 10.3390/plants11111416. 35684189 10.3390/plants11111416 PMC9183004

[85] Fusco GM, Campana E, Ciriello M, Nicastro R, De Paola F, De Tommaso G, et al. Compost and biochar synergy enhances photosynthetic efficiency and antioxidant responses in leafy vegetables under sub-optimal nutrient supply. Plant Stress. 2026;19:101244. 10.1016/j.stress.2026.101244.

[86] Helaoui S, Boughattas I, Mkhinini M, Chebbi L, Elkribi-Boukhris S, Alphonse V, et al. Biochar amendment alleviates heavy metal phytotoxicity of Medicago sativa grown in polymetallic contaminated soil: Evaluation of metal uptake, plant response and soil properties. Plant Stress. 2023;10:100212. 10.1016/j.stress.2023.100212.

[87] Qaisi AM, Al Tawaha AR, Imran A-R. Effects of chitosan and biochar-mended soil on growth, yield and yield components and mineral composition of fenugreek. Gesunde Pflanzen. 2023;75:625–36. 10.1007/s10343-022-00727-x.

[88] El Nahhas N, AlKahtani MDF, Abdelaal KAA, Al Husnain L, AlGwaiz HIM, Hafez YM, et al. Biochar and jasmonic acid application attenuates antioxidative systems and improves growth, physiology, nutrient uptake and productivity of faba bean (*Vicia faba* L.) irrigated with saline water. Plant Physiol Biochem. 2021;166:807–17. 10.1016/j.plaphy.2021.06.033. 34225005 10.1016/j.plaphy.2021.06.033

[89] El-Hassanin A, Samaka M, El-Hady O, El-Dewiny C, Mostafa F. Impact of biochar and hydrogel amendments on hydrophysical properties of sandy soil and cowpea yield (*Vigna Unguiculata* L.) under different water regimes. Egypt J Chem. 2022; 65: 487–497. 10.21608/ejchem.2021.97083.4542

[90] Velli P, Manolikaki I, Diamadopoulos E. Effect of biochar produced from sewage sludge on tomato (*Solanum lycopersicum* L.) growth, soil chemical properties and heavy metal concentrations. J Environ Manage. 2021;297:113325. 10.1016/j.jenvman.2021.113325. 34325369 10.1016/j.jenvman.2021.113325

[91] Lentini M, Ciriello M, Carillo P, Fusco GM, Nicastro R, Pannico A, et al. Enhancing San Marzano dwarf tomato performance: The role of biochar under saline irrigation conditions. Plant Stress. 2026;19:101184. 10.1016/j.stress.2025.101184.

[92] Santos MGBD, Paiva AB, Costa CR, Paiva MB, Mendes GDO, Martins ÉDS, et al. The role of oxalic acid in nutrient solubilization, potentially toxic elements from sewage sludge biochar, and remineralizers for sustainable local fertilizers. Environ Res. 2026;294:123818. 10.1016/j.envres.2026.123818. 41577109 10.1016/j.envres.2026.123818

[93] Farid IM, Siam HS, Abbas MHH, Mohamed I, Mahmoud SA, Tolba M, et al. Co-composted biochar derived from rice straw and sugarcane bagasse improved soil properties, carbon balance, and zucchini growth in a sandy soil: A trial for enhancing the health of low fertile arid soils. Chemosphere. 2022;292:133389. 10.1016/j.chemosphere.2021.133389. 34953878 10.1016/j.chemosphere.2021.133389

[94] Seleiman MF, Refay Y, Al-Suhaibani N, Al-Ashkar I, El-Hendawy S, Hafez EM. Integrative effects of rice-straw biochar and silicon on oil and seed quality, yield and physiological traits of *Helianthus annuus* L. grown under water deficit stress. Agronomy (Basel). 2019;9:637. 10.3390/agronomy9100637.

[95] Hafez EM, Gowayed SM, Nehela Y, Sakran RM, Rady AMS, Awadalla A, et al. Incorporated biochar-based soil amendment and exogenous glycine betaine foliar application ameliorate rice (*Oryza sativa* L.) tolerance and resilience to osmotic stress. Plants (Basel, Switzerland). 2021;10:1930. 10.3390/plants10091930. 34579461 10.3390/plants10091930 PMC8471834

[96] Baronti S, Vaccari FP, Miglietta F, Calzolari C, Lugato E, Orlandini S, et al. Impact of biochar application on plant water relations in *Vitis vinifera* (L.). Eur J Agron. 2014;53:38–44. 10.1016/j.eja.2013.11.003.

[97] Iboko MP, Dossou-Yovo ER, Témé N, Obalum SE, Diedhiou S, Konan AKMS, et al. Combining biochar, nitrogen fertilizer and no-tillage to reduce greenhouse gas emissions and increase rice yield in rainfed lowland. J Agric Food Res. 2026;26:102700. 10.1016/j.jafr.2026.102700.

[98] Guan R, Li Y, Jia Y, Jiang F, Li L, Biswas A, et al. Dual impact of single acidified biochar application on saline-alkaline soil: short-term salinization risks and persistent nutrient benefits. Soil Tillage Res. 2025;254:106745. 10.1016/j.still.2025.106745.

